# Botulinum toxin injection strategy of intractable and relapsed piriformis syndrome: A case report

**DOI:** 10.1097/MD.0000000000030950

**Published:** 2022-10-21

**Authors:** So Young Kwon, Eun Hwa Jun, Seong Jin Park, Yumi Kim

**Affiliations:** Department of Anesthesiology and Pain Medicine, St. Vincent Hospital, College of Medicine, The Catholic University of Korea, Suwon, South Korea.

**Keywords:** back pain, botulinum toxin, case report, fluoroscopic guided, piriformis syndrome, ultrasound guided

## Abstract

Piriformis syndrome (PS) is neuromuscular disorder caused by sciatic nerve compression by piriformis muscle and related to sciatic-type pain. When the conservative care fails, local injection or surgery can be also performed into piriformis. In recent years, botulinum toxin (BoNT) has also been considered as a new therapeutic option of piriformis syndrome.

**Patient concerns:**

A man in his late 40s came to pain clinic for left low back pain. The symptom was aggravated with sitting position.

**Diagnosis:**

Piriformis syndrome.

**Interventions:**

The patient underwent BoNT injection with 100 IU with 2 mL into piriformis muscle for piriformis syndrome treatment, and his pain was relieved. However, it recurred 8 months later. BoNT injection was repeated with 100 IU with 5 mL.

**Outcomes:**

At the time of this writing, his pain was reduced for 2 years without any medication.

**Lessons:**

We report a case of treating relapsed piriformis syndrome with BoNT injection of different dilution volume, suggesting that the higher the dilution volume, the more effective for therapeutic effect of BoNT.

## 1. Introduction

Piriformis syndrome (PS) is neuromuscular disorder caused by sciatic nerve compression by piriformis muscle, and related to sciatic-type pain, tingling and numbness in the buttocks and lower thigh. The pain is often aggravated by prolonged sitting or rising from a seated position.

Conservative therapeutic strategies of PS include stretching exercises, massage, physical therapies (heat, ultrasound, laser therapy), and medication (nonsteroidal anti-inflammatory drugs, analgesics, and muscle relaxants). When the conservative care fails, local injection of piriformis muscle with or without corticosteroid or surgery can be also performed into piriformis.^[1]^

Botulinum toxin (BoNT) has also been considered as a therapeutic option of PS. Several studies showed that BoNT injection led to improvement in pain and a decrease in the thickness and volume of the muscle.^[2–4]^ BoNT is used for focal hypertonia that works by hindering the release of acetylcholine at the motor endplates of the targeted muscle, and reduce spasticity, dystonia, and related disorders.

For accuracy and therapeutic effect of BoNT injection, various types of image-guided injection are used, and research is also active. However, there are no established dilutional protocols or guideline, and there are variations among physicians in the amount of saline.^[5]^ We report a case of different dilution volume of BoNT injection into piriformis of a patient with intractable and relapsed piriformis syndrome.

## 2. Case

A man in his late 40s came to pain clinic for left low back pain. The symptom was started 1 year ago and aggravated 2 months ago. He couldn’t walk even 10 m, when the pain was severe, and the pain was radiated down to left buttock, ankle and dorsum of the foot, and it was exacerbated with prolonged sitting position. Pain score was measured by numeric rating scale of pain (NRS), and NRS in sitting position was 8. He had a job spending much time with sitting position. He had limitation of motion with severe pain, and tenderness of left piriformis muscle.

He had hypertension, and no specific family and psychosocial history associated with symptoms.

Physical examination revealed no neurologic sign of lumbar herniated disc or cord compression and positive for flexion, adduction, internal rotation (FAIR) test and Freiberg’s maneuver, suggesting PS.

Lumbar spine X-ray showed spondylosis with narrowing of Lumbar 4‐5 disc space and mild spondylolisthesis lumbar 4 on 5.

Medical treatment with gabapentin 300 mg/day, anti-inflammatory analgesic drug (zaltoprofen), and muscle relaxant for 4 weeks did not lead to pain improvement. Caudal block with lidocaine was also performed twice to exclude spine origin, but it was ineffective.

**Figure 1.**
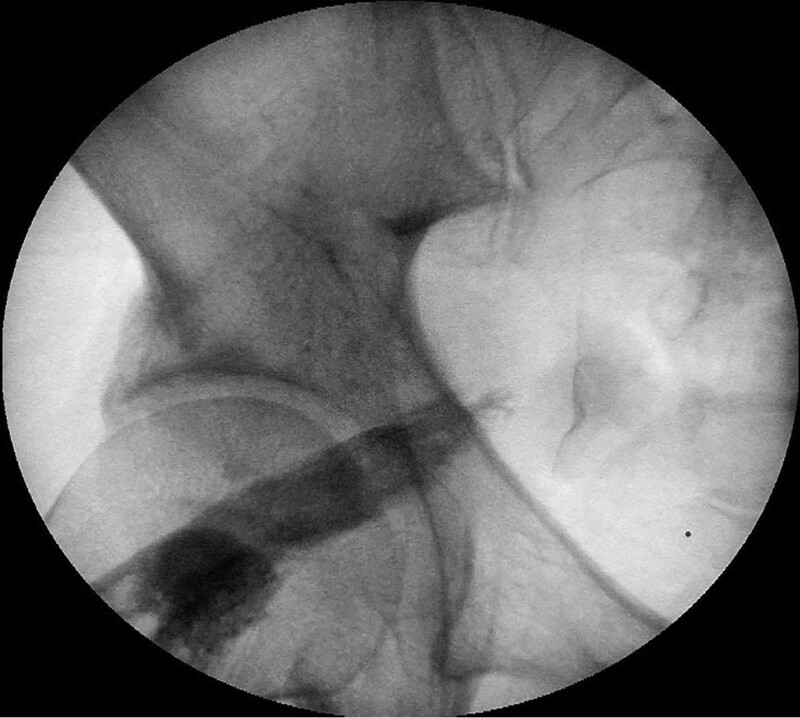
Fluoroscopic image of the piriformis muscle after contrast injection.

Under ultrasound guided, BoNT injection was performed with 100 IU of BoNT type A with 2 mL of saline dilution. Before the procedure, informed content to treatment was obtained. BOTOX (Allergan plc, Dublin, Ireland) was used. With prone position, under the ultrasound, the piriformis muscle was identified between gluteal muscle and ischial spine. The needle was inserted in plane with ultrasound transducer until it enters the piriformis muscle tissue, and the needle placement was confirmed again with contrast injection under fluoroscopy before injecting medication (Fig. 1). After the injection, pain was reduced to NRS 3, and medication was maintained with same dose for 2 weeks.

Eight months later, he came to clinic again with relapsed pain. He was almost pain-free during his absence from the hospital and was not on medication. The relapsed pain score was NRS 7, and the symptom was aggravated with sitting position. After ultrasound guided local anesthetics injection (2% lidocaine 2mL with 8 mL of saline) to left piriformis muscle, the pain was resolved, but the effect lasted only 2–3 days. Medication was started with pregabalin 150 mg/day, amitriptyline 5 mg, tianeptine 25 mg.

**Figure 2.**
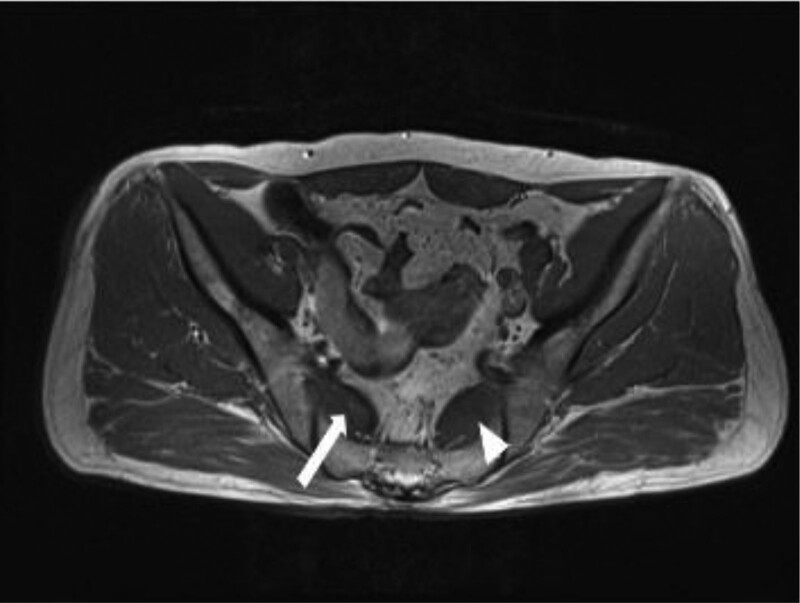
MRI axial T1-weighted image showing asymmetry of the piriformis muscles, with hypertrophy of the left piriformis muscle (white arrow) than right (white arrowhead).

Magnetic resonance image (MRI) showed hypertrophy of left piriformis muscle, but there was no evidence of sciatic nerve neuropathy (Fig. 2).

**Figure 3.**
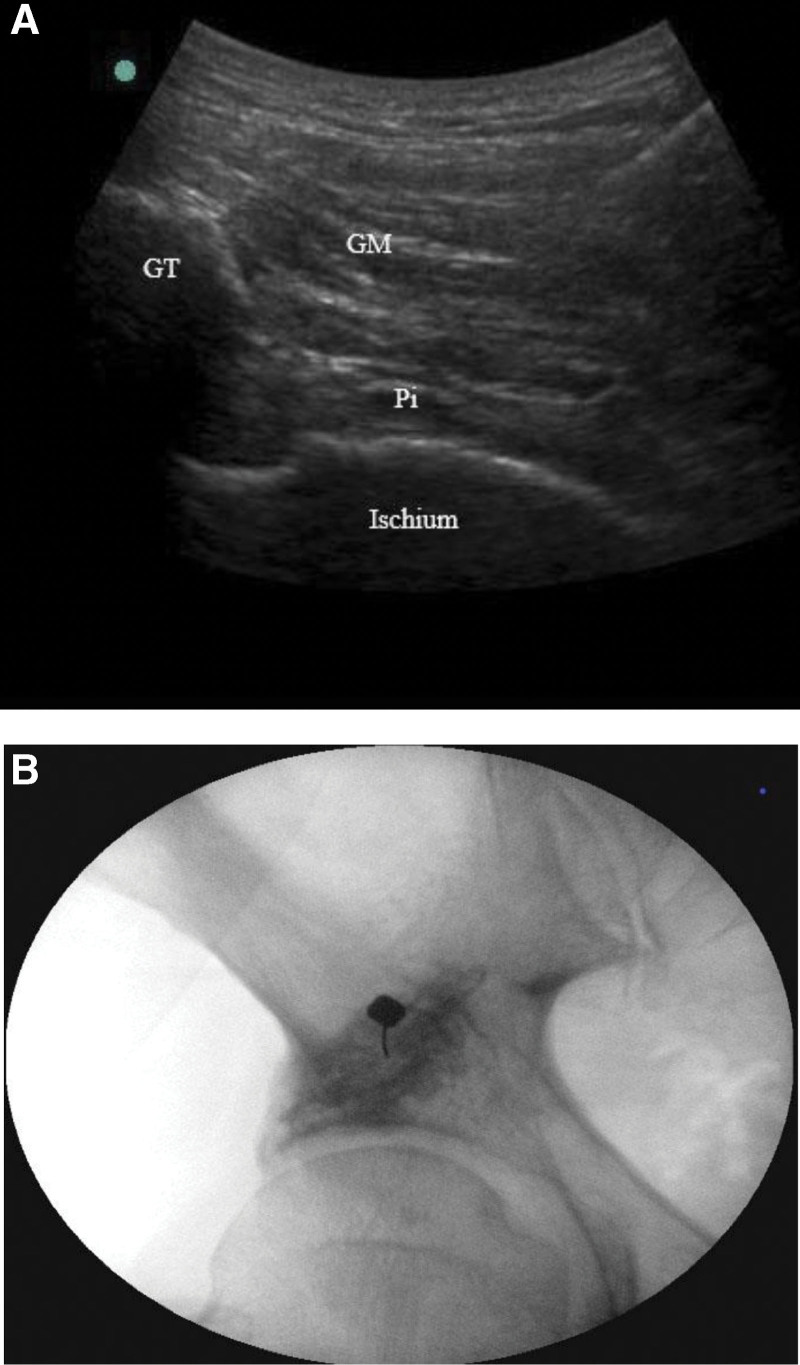
(A) Ultrasound image of the left buttock during targeted piriformis injection. (B) Fluoroscopic image of the piriformis muscle after contrast injection. GM = gluteus maximus, GT = greater tuberosity, Pi = piriformis.

We repeated BoNT injection in the left piriformis with 100 IU of BoNT with 5 mL of saline dilution. In the same way as in the previous injection, the needle was inserted using ultrasound guidance, and the placement was confirmed with contrast injection under fluoroscope. (Fig. 3) There was no complication after procedure. Medication was maintained with same dose for 4 weeks. In a few weeks after the injection, the pain was relieved, and it has remained without symptoms for 2 years.

## 3. Discussion

PS may be caused by anatomic abnormalities of the piriformis muscle and the sciatic nerve resulting in irritating of the sciatic nerve. Patients with PS often present with hip pain, buttock pain, dyspareuria (in female patients), and sciatica. The pain is often aggravated by prolonged sitting or rising from a seated position.

There is no gold standard test for PS, and the diagnosis is usually made after exclusion of other possibility of sciatic pain such as spinal stenosis, facet syndrome, and sacroiliac joint dysfunction.^[6]^ Several clinical signs can be identified by specific tests during physical examination, particularly if the patient presents with sciatic pain without concomitant lower back pain, and depending on position.

Several physical examinations help diagnose piriformis syndrome, such as Freiberg maneuver, FAIR test, active piriformis test, Beatty test, and Pace test. In the Freiberg maneuver and FAIR test, typical pain in the posterior pelvis or paresthesia represents a positive finding. A positive Pace or Beatty test is indicated buttock pain, weakness, or paresthesia on affected side.

Imaging study can be helpful to assist the diagnosis, because it can show structural changes in the muscle, and help to exclude other possibilities. MRI can identify anatomical abnormalities or side-to-side difference of piriformis muscle, but piriformis muscle enlargement on MRI has been reported in 19% of asymptomatic patients.^[7]^ In addition, ultrasound can visualize myofascial trigger points in piriformis muscle with increased resting tone.

In this case, at first, we diagnosed PS empirically base on only his history and physical examination. After the first BoNT injection failed, muscle hypertrophy and trigger point were directly confirmed through MRI and ultrasound.

The management of PS includes conservative care, injection of the piriformis muscle with local anesthetics and steroid, or BoNT. Piriformis injections are often performed diagnostically and therapeutically to assist in the treatment of patient with buttock and leg pain.

In recent years, BoNT injections have shown improvement in pain compared of local anesthetics.^[8,9]^

BoNT is used for focal hypertonia that works by hindering the release of acetylcholine at the motor endplates of the targeted muscle. The therapeutic effects are acknowledged to be relieving muscle contractions and stopping the pain cycle.^[10]^

The most common adverse effect of BoNT injection is prolonged pain. Foster et al reported that it assumed that incorrectly injecting into the tendinous portion or nerve cause tendinitis and mechanical nerve damage.^[11]^

Therefore, Injection accuracy is an important factor for therapeutic effectiveness, but it is difficult to access of the needle because of deep location and small size of the muscle. Several techniques of piriformis injections have been proposed: Computer Tomography, MRI, ultrasound, fluoroscopy, or, electrical stimulators.

Ultrasound has been reported to be superior to fluoroscopy, and in a previous cadaveric study, only 30% of fluoroscopically placed injections were accurate, compared with 95% of ultrasound-guided injections by an experienced clinician.^[12]^

Fluoroscopy guided injection is using the greater trochanter of the femur and lateral border of sacrum and sacroiliac joint as landmarks. Placement of needle is confirmed by contrast. Golzalez et al^[13]^ reported the cadaveric study that the needle placement within piriformis muscle in fluoroscopic-guided injection is lateral to sciatic nerve location.

Ultrasound-guided injections are also widely used because of their reliability, simplicity, and absence of ionizing radiation, but they have some disadvantages such as user dependent and time of provider in clinic to perform procedure. Especially, ultrasound needs experienced provider, and we confirmed again with contrast injection for the accuracy of injection site.

Therefore, in this case by combining the advantages of both imaging tools, the accuracy of the injection was improved. We believe that imaging the muscle hypertrophy and injecting in a more precise location helped to increase the therapeutic effect of BoNT injection.

The efficacy of BoNT may be determined by not only accuracy of injection, but also the physical spread of the molecule from the injection site, passive diffusion. Volume and dilution may also influence diffusion and spread of BoNT.^[14]^ There are no established dilution protocol and guidelines, and there are great variations among physicians in the amount of saline. Several studies reported that increasing dilution volume may result in greater diffusion and increase the beneficial of BoNT effect.^[15,16]^ One possible explanation is recruitment of more motor units due to the enhanced spread of toxin with a higher dilution volume. In this case, we injected 100U of BoNT diluted with 2 mL and 5 mL, and a higher dilution volume had a longer therapeutic effect. Low concentration and higher volume of BoNT might be greater diffusion and larger affected area.

Our limitation is that only one case is reported. In this one case alone, it may seem that the difference in the concentration of BoNT is not the only factor that determines the effectiveness of the treatment. However, there was no difference in the injection method, and voluntary rehabilitation, or the work environment of the patent were not changed. It is necessary to consider the effect of the dilution volume on the therapeutic effect and, further investigation with larger sample should be carried out. Although it is difficult to present an appropriate dilution concentration in this case, low concentration, and higher volume of BoNT might be greater diffusion and larger affected area.

## 4. Conclusions

In conclusion, we suggest that increasing the dilution volume might be more effective for the therapeutic effect of BoNT.

## Author contributions

**Conceptualization:** So Young Kwon.

**Data curation:** Seong Jin Park.

**Investigation:** Seong Jin Park.

**Supervision:** Yumi Kim.

**Project administration:** So Young Kwon.

**Visualization:** Eun Hwa.

**Writing – original draft:** So Young Kwon.

**Writing – review & editing:** Yumi Kim.
